# Association Between Mutation Context–Associated RUNX1 Transcriptional States and Immune–Stromal Heterogeneity in Nonsmall Cell Lung Cancer

**DOI:** 10.1155/humu/6216101

**Published:** 2026-09-06

**Authors:** Jinyao Wang, Shuang Li, Jingjing Zhou, Jing Yang, Huan Ma

**Affiliations:** ^1^ Department of Gerontology, The First Affiliated Hospital of Jinzhou Medical University, Jinzhou, China, jzmu.edu.cn; ^2^ Department of Oncology, The First Affiliated Hospital of Jinzhou Medical University, Jinzhou, China, jzmu.edu.cn; ^3^ Department of Chronic Disease Management Center, The First Affiliated Hospital of Jinzhou Medical University, Jinzhou, China, jzmu.edu.cn; ^4^ Department of Pathology, The First Affiliated Hospital of Jinzhou Medical University, Jinzhou, China, jzmu.edu.cn

**Keywords:** mutation context, nonsmall cell lung cancer, RUNX1, spatial transcriptomics, target-gene-set activity, transcriptome-based immune scoring, tumor microenvironment

## Abstract

Nonsmall cell lung cancer (NSCLC) is characterized by substantial heterogeneity in driver mutation backgrounds and tumor microenvironment (TME) phenotypes, yet the transcriptional regulatory states linking genotype context to immune–stromal variation remain incompletely defined. In this study, we integrated mutation, transcriptomic, and clinical data from TCGA‐LUAD/LUSC to construct a mutation–expression‐matched NSCLC cohort and evaluated RUNX1 as a mutation context–associated transcriptional/regulatory state rather than as a recurrently mutated driver. Driver contexts were assessed using a prespecified six‐gene panel comprising EGFR, KRAS, TP53, STK11, KEAP1, and BRAF in the overall NSCLC cohort, the primary LUAD analysis, and a descriptive LUSC sensitivity analysis. The prespecified driver‐context‐negative operational group was defined as tumors without mutations in any of these six genes. RUNX1 mutation status was reported separately and did not contribute to this definition. Analyses included RUNX1 distributions, six‐gene overlap, the RUNX1 cutoff, comutation burden, model sample‐size audits, and adjusted context models. RUNX1 expression and inferred RUNX1 target‐gene‐set activity were evaluated across mutation contexts, whereas TME features were characterized using MCP‐counter, xCell, ssGSEA, and ESTIMATE. Single‐cell RNA sequencing and Visium spatial transcriptomic datasets were further used to localize RUNX1 expression, inferred target projections, and candidate TME‐supporting genes. RUNX1 was prioritized within a prespecified candidate‐screening framework and was significantly associated with the inferred RUNX1 target‐gene‐set readout. RUNX1 expression differences required interpretation in relation to histology and comutation structure. In the primary LUAD analysis, the KRAS‐mutant context was associated with higher RUNX1 expression, whereas the KEAP1‐mutant context was associated with lower expression. LUSC findings were retained as a descriptive sensitivity analysis and were not given the same inferential weight. Six‐gene overlap, comutation burden, the RUNX1 cutoff, and model sample‐size audits further showed that driver labels were nonmutually exclusive and that the prespecified driver‐context‐negative group was an operational reference rather than a biologically homogeneous subtype. RUNX1‐high and RUNX1‐low tumors displayed mutation context–dependent immune and stromal differences, involving stromal/cancer‐associated fibroblast, myeloid/dendritic cell, endothelial, immune‐score, and tumor‐purity axes. Single‐cell analysis localized RUNX1‐associated signals and target‐gene projections across epithelial‐like and myeloid compartments, and spatial transcriptomics supported concordance between RUNX1 and selected TME‐supporting genes in NSCLC tissue sections. Public immune checkpoint inhibitor cohorts were limited by small sample size and incomplete annotations and did not support RUNX1 as a stable immunotherapy‐response predictor. Overall, these findings propose a mutation‐context‐aware RUNX1–TME stratification framework and generate testable hypotheses for mechanistic validation and mutation‐annotated immunotherapy studies.

## 1. Introduction

Nonsmall cell lung cancer (NSCLC) is one of the most common types of lung cancer. Lung adenocarcinoma (LUAD) and lung squamous cell carcinoma (LUSC) differ substantially in driver mutation spectra, transcriptomic states, transcriptome‐based immune scores, and therapeutic responses [1, 2]. Driver mutation contexts represented by EGFR, KRAS, TP53, STK11, and KEAP1 have been reported to be associated with tumor cell states and TME features such as immune exclusion, myeloid enrichment, stromal‐associated expression changes, antigen presentation, and immune checkpoint status [3–7]. Previous studies have often used the expression of a single gene or the abundance of a single immune cell type as a surrogate for tumor immune status. However, in highly heterogeneous tumors such as NSCLC, single‐gene or single‐axis analyses that are detached from the driver mutation context often fail to explain the complex immune–stromal differences observed among patients.

RUNX1 is an important transcription factor involved in hematopoiesis, development, and tumor transcriptional regulation. In multiple cancers, RUNX1 can participate in tumor progression by regulating cellular differentiation, inflammatory signaling, and cell‐state transitions [8, 9]. However, in NSCLC, RUNX1 itself is not a frequently recurrently mutated gene. Observations based on public cBioPortal data also indicate that its mutation frequency is low, with no clear hotspot mutations or prominent copy‐number alterations [10, 11]. Previous NSCLC studies have suggested that RUNX1 is associated with clinicopathological characteristics, methylation/expression status, and cell migration [12, 13]. Therefore, the evidence base for studying RUNX1 as a mutational driver gene in NSCLC is insufficient. Instead, RUNX1 is more likely to act as a downstream transcriptional state or target‐gene‐set state under different driver mutation contexts, contributing to the interpretation of tumor cell states and microenvironmental differences. In other words, the research value of RUNX1 may not lie in “RUNX1 mutation” itself, but rather in whether it can serve as a mutation context–associated transcriptional interpreter of the relationship between genotype context and TME phenotype.

Based on this rationale, we constructed a mutation context–aware analytical framework using a TCGA‐LUAD/LUSC mutation–expression‐matched cohort as the primary discovery layer, together with public immune checkpoint inhibitor (ICI) cohorts, single‐cell RNA sequencing data, and spatial transcriptomic data. This framework was used to systematically evaluate changes in RUNX1 expression and inferred RUNX1 target‐gene‐set activity across different driver mutation contexts and to further characterize their relationships with immune/stromal microenvironmental features. Our goal was not to demonstrate that RUNX1 directly alters the immune–stromal ecosystem nor to establish a clinical prediction model. Rather, we aimed at proposing a testable framework for organizing and interpreting the exploratory associations among genotype context, RUNX1‐associated transcriptional/target‐gene‐set state, and TME heterogeneity.

## 2. Materials and Methods

### 2.1. Data Sources and Study Cohorts

This study used TCGA‐LUAD and TCGA‐LUSC as the primary discovery cohorts, integrating mutation data, bulk RNA‐seq expression profiles, and clinical survival information [1, 2]. The main upstream datasets used in the project workspace included TCGA‐LUAD/LUSC FPKM expression matrices, clinical tables, survival tables, and an NSCLC mutation–expression table with integrated RUNX1 expression. A sample‐level analysis matrix integrating mutation context, RUNX1 expression, ESTIMATE scores, and inferred RUNX1 target‐gene‐set scores is provided in Table S1. Public immunotherapy cohorts, including GSE135222 and GSE126044, were used to explore the association between RUNX1 and ICI response [14–16]. Single‐cell analysis was performed using GSE207422 NSCLC scRNA‐seq raw counts and corresponding metadata [17]. Spatial transcriptomic analysis used E‐MTAB‐13530 NSCLC Visium data [18], which were reused in a read‐only manner from an existing legacy workspace.

All public datasets were organized according to their original databases and project file records. The present study did not modify upstream data in the legacy workspace. Only derived tables, figures, and manuscript files were generated in the RUNX1_MutTME workspace.

### 2.2. Definition of Driver Mutation Contexts

**Figure 1:**
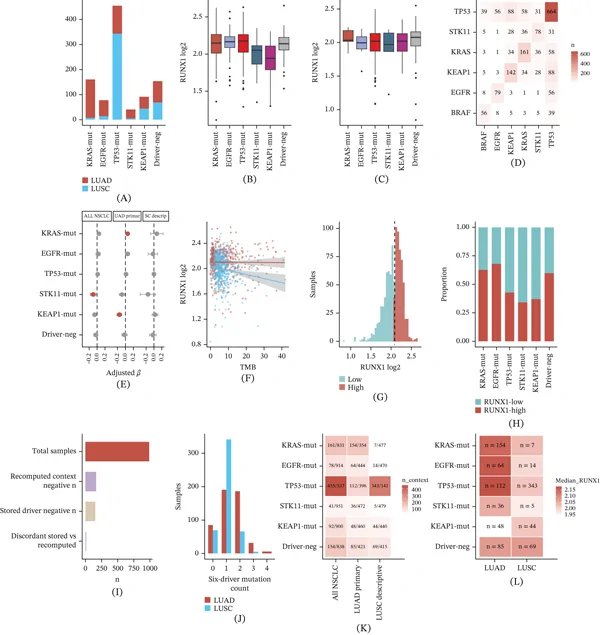
Mutation‐context definitions and histology‐aware models of RUNX1 expression. This figure defines the prespecified mutation‐context framework and evaluates associations between mutation context and RUNX1 expression using histology‐stratified distributions, six‐gene overlap, comutation burden, TMB, covariate‐adjusted models, and definition and sample‐size audits. (A) Histologic composition of the five displayed mutation‐positive contexts and the prespecified driver‐context‐negative group. Context classification was based on six prespecified genes, EGFR, KRAS, TP53, STK11, KEAP1, and BRAF. (B) Distribution of log2‐transformed RUNX1 expression across the displayed mutation contexts in LUAD, which served as the primary histology‐aware analysis layer. (C) Distribution of log2‐transformed RUNX1 expression across the displayed mutation contexts in LUSC. Because several LUSC mutation contexts contained few samples, this layer was interpreted as a descriptive sensitivity analysis.(D) Six‐gene overlap matrix showing the numbers of samples shared between pairs of prespecified context genes. The matrix illustrates that the driver labels were nonmutually exclusive and that mutation‐positive versus remaining‐sample comparisons were not clean wild‐type contrasts. (E) Covariate‐adjusted context‐model coefficients with 95% confidence intervals for all NSCLC, LUAD, and LUSC. The all‐NSCLC model included histology, continuous TMB, estimated tumor purity, and six‐gene comutation burden. Histology was omitted as a covariate in the LUAD‐ and LUSC‐specific models. (F) Relationship between continuous TMB and log2‐transformed RUNX1 expression, with LUAD and LUSC displayed separately. This analysis was treated as a mutation‐burden companion analysis. (G) Distribution of log2‐transformed RUNX1 expression showing the analysis‐eligible TCGA cohort median cutoff of approximately 2.085. The cutoff was used for exploratory RUNX1‐low and RUNX1‐high grouping and was not defined as a clinical threshold. (H) Proportions of RUNX1‐low and RUNX1‐high samples within the displayed mutation contexts.(I) Driver‐negative definition quality‐control analysis comparing the total sample count, stored driver‐negative classification, recomputed six‐gene context‐negative classification, and discordant stored versus recomputed assignments. The prespecified driver‐context‐negative group comprised samples without detected mutations in EGFR, KRAS, TP53, STK11, KEAP1, or BRAF. RUNX1 mutation status was not used in this definition, and the group was treated as an operational reference rather than a biologically homogeneous subtype. (J) Distribution of the number of mutated prespecified context genes per sample, stratified by LUAD and LUSC, illustrating the six‐gene comutation burden. (K) Model sample‐size audit reporting n_context/n_reference for each displayed context in the all‐NSCLC, LUAD‐primary, and LUSC‐descriptive models. (L) Median RUNX1 expression and sample size for each displayed context after stratification by LUAD and LUSC. This figure supports histology‐aware, comutation‐aware, and covariate‐adjusted associations between mutation context and RUNX1 expression. It does not demonstrate direct regulation of RUNX1 by driver mutations, establish the prespecified driver‐context‐negative group as a biologically homogeneous subtype, or define the median RUNX1 cutoff as a clinical threshold.

NSCLC samples were stratified according to prespecified driver mutation contexts. Figure 1 uses six prespecified context genes, EGFR, KRAS, TP53, STK11, KEAP1, and BRAF, as a common denominator and presents context sample counts in the overall NSCLC cohort, RUNX1 distributions in the primary LUAD analysis, a descriptive LUSC sensitivity analysis, six‐gene overlap, comutation burden, a model sample‐size audit, and context‐level median RUNX1 expression. The prespecified driver‐context‐negative operational group was defined as samples without mutations detected in any of these six genes. RUNX1 mutation status was reported separately as an observed event and was not used to define this group. The stored classification was compared with a classification recomputed from the absence of mutations in the six prespecified genes, and the corresponding definition audit and group‐level quality‐control summaries are provided in Tables S2 and S3. This operational group served as a reproducible reference group and was not intended to represent either a biological subtype lacking any oncogenic driver or a homogeneous molecular subtype. Mutation data processing and visualization followed the MAF/maftools framework [19].

All driver labels were treated as nonmutually exclusive descriptive labels, and the same sample could belong to multiple driver‐positive or comutation contexts. Accordingly, single‐driver analyses did not constitute comparisons against strictly defined wild‐type groups. In addition to the single‐driver layer, we further analyzed comutational combinations, including KRAS + TP53, KRAS + STK11, and STK11 + KEAP1. TMB tertiles and continuous TMB were incorporated as companion mutation‐burden analyses. LUAD was prioritized for primary interpretation, LUSC was used as a descriptive sensitivity analysis, and pooled all‐NSCLC results provided the cohort‐level overview. RUNX1‐high and RUNX1‐low groups were defined using the median in the analysis‐eligible TCGA cohort, corresponding to a RUNX1 log2 cutoff of approximately 2.085. Sample counts stratified by histology, stored driver‐context category, and RUNX1 group are provided in Table S4. Where feasible, continuous RUNX1 or continuous context‐model readouts were also reported to avoid interpreting median‐based grouping as a clinical threshold.

When the sample size of a given mutation context or combined subgroup was insufficient, the corresponding results were presented only as direction‐of‐effect or exploratory findings, thereby avoiding the interpretation of underpowered results as strong conclusions.

### 2.3. RUNX1 Expression and Target‐Gene‐Set Scores

RUNX1 expression was quantified using log2‐transformed expression values from bulk RNA‐seq data. To evaluate whether RUNX1 expression corresponded to transcription factor target‐gene‐set readouts, we used inferred target‐gene‐set scores based on a RUNX1 target set, including ULM scores and AUCell‐style scores. The corresponding target set comprised 18 confidence‐A signed RUNX1 targets derived from DoRothEA, with reference to the DoRothEA, CollecTRI, decoupleR, and SCENIC/AUCell frameworks [20–23]. It should be emphasized that the single‐cell‐level RUNX1 target projection in the present study represents a transparent target‐gene projection analysis and does not constitute complete pySCENIC‐based target‐gene‐set validation. Therefore, throughout the manuscript, we refer to these results as “inferred RUNX1 target‐gene‐set activity” or “target projection,” without upgrading them to evidence of protein‐binding mechanisms, complete gene regulatory network proof, or direct validation of target‐gene regulation.

### 2.4. Three‐Tier Mutational Context Analysis

At the single‐driver level, we compared RUNX1 expression between each driver context and the corresponding reference samples. Hedges′s *g* was used to measure effect size, whereas Wilcoxon tests and the Benjamini–Hochberg (BH) procedure were used for statistical testing and multiple‐testing correction. Figure 1 further separated this layer into a primary LUAD analysis and a descriptive LUSC sensitivity analysis to avoid a simple pooled interpretation of LUAD‐enriched driver contexts and the LUSC‐enriched TP53 context. At the comutation level, we compared RUNX1 expression states across prespecified comutational combinations and recorded the six‐gene mutation count and overlap matrix. At the TMB level, samples were grouped by tertiles, and nonparametric tests and Spearman correlation analysis were used to evaluate the relationship between RUNX1 expression and mutation burden.

To address potential confounding by histology, comutation burden, TMB, and estimated tumor purity, adjusted context models were reported in Figure 1E. The all‐NSCLC model included histology, TMB, estimated tumor purity, and six‐gene comutation burden. The primary LUAD and descriptive LUSC subset models did not include histology as a covariate. Model outputs included adjusted beta coefficients, 95% confidence intervals, *p* values, BH‐FDR values, context sample sizes, and reference sample sizes (Table S5). Figure 1K provides a sample‐size audit showing the estimability of each context in the all‐NSCLC, primary LUAD, and descriptive LUSC strata.

### 2.5. Multialgorithm Immune and Stromal Microenvironment Scoring

TME analysis used four categories of transcriptome‐based immune/stromal scoring outputs that were available in the current workspace: MCP‐counter, xCell, ssGSEA immune signatures, and ESTIMATE [24–28]. CIBERSORT/CIBERSORTx, EPIC, quanTIseq, and TIMER were not used as executed primary evidence because output tables from these algorithms were not present in the current workspace. Outputs from each algorithm were mapped to immune/stromal axes, including CD8/cytotoxic, T cell, B cell, myeloid, myeloid/dendritic cell (DC), stromal/cancer‐associated fibroblast (CAF), endothelial, immune score, and tumor purity.

**Figure 2:**
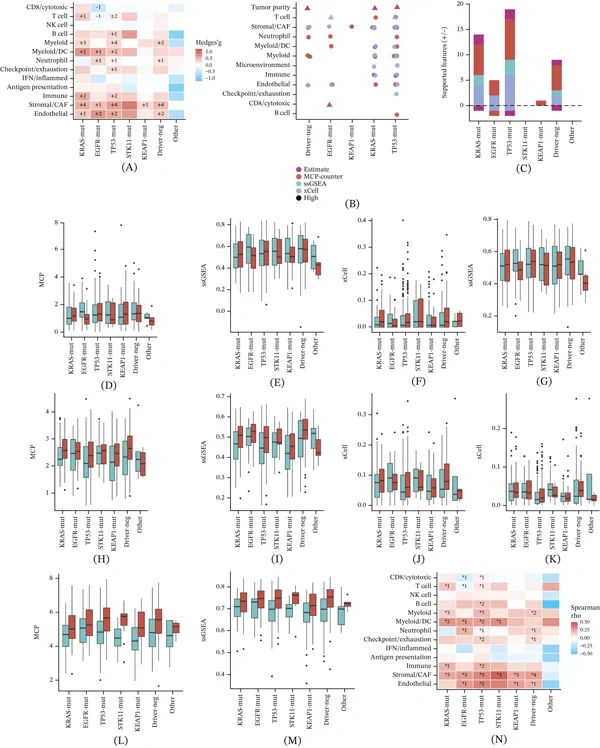
Multialgorithm immune and stromal microenvironment landscape associated with mutation context and RUNX1 state. Using MCP‐counter, xCell, ssGSEA immune signatures, and ESTIMATE scores derived from the same TCGA bulk RNA‐seq matrix, this figure characterizes multiaxis transcriptomic TME variation associated with mutation context × RUNX1 state. (A) Immune‐axis effect‐size matrix summarizing the direction and magnitude of differences between RUNX1‐high and RUNX1‐low tumors across mutation contexts for CD8/cytotoxic, T cell, NK cell, B cell, myeloid, myeloid/DC, neutrophil, checkpoint/exhaustion, IFN/inflamed, antigen‐presentation, immune, stromal/CAF, endothelial, tumor‐purity, and related axes. Cell annotations indicate the number and direction of supported feature‐level results. (B) Supported algorithm‐hits dot plot showing context–feature combinations that reached directional and statistical support, together with their algorithmic sources. (C) Stacked counts of positively and negatively supported features within each mutation context, stratified by algorithm. (D) Distribution of MCP‐counter CD8 T‐cell scores across contexts and RUNX1 groups. (E) Context‐stratified distribution of the ssGSEA cytolytic score. (F) Context‐stratified distribution of xCell CD8 T‐cell scores. (G) Context‐stratified distribution of the IFN‐*γ* signature. (H) Distribution of MCP‐counter monocyte scores. (I) Distribution of the myeloid‐suppression signature. (J) Distribution of xCell macrophage scores. (K) Distribution of xCell macrophage M2 scores. (L) Distribution of MCP‐counter CAF scores. (M) Distribution of the CAF signature. (N) Correlation heatmap between RUNX1 expression and immune/TME features across mutation contexts. Because all four scoring approaches were applied to the same TCGA bulk RNA‐seq matrix, crossalgorithm agreement represents methodological consistency within a shared data source and does not constitute independent immune validation. CIBERSORT/CIBERSORTx, EPIC, quanTIseq, and TIMER did not generate usable outputs in the current workspace and therefore should not be described as executed evidence. This figure emphasizes multialgorithm, multiaxis transcriptomic TME associations but does not establish changes in immune‐cell abundance or recruitment, causal immune remodeling, or direct reshaping of immune‐cell composition by RUNX1.

For each mutation context, two types of analyses were performed. First, TME score differences between RUNX1‐high and RUNX1‐low samples were compared, with Hedges′s *g*, Wilcoxon *p* values, BH‐FDR values, and underpowered flags reported. Second, purity‐aware linear models using continuous “RUNX1_log2” as the predictor were fitted. The model was specified as “score ~ RUNX1_log2 + cancer_type_label + TMB + Estimated_purity_cov + co_mutation_count”. The “cancer_type_label” term was omitted in single‐histology subsets. These models provided the adjusted coefficient readouts presented in Figure 2B and Table S6. Corresponding axis‐level summaries are provided in Table S7. ESTIMATE tumor purity was displayed as “Tumor_purity” in the figures, whereas the separate variable “Estimated_purity_cov” was used as a model covariate. Interpretation at the multialgorithm level emphasized associations and consistency within the same TCGA bulk matrix. These findings were not treated as independent immune validation or as evidence of immune‐cell recruitment or causal TME remodeling.

### 2.6. Exploratory Analysis of ICI Cohorts

The public ICI cohorts GSE135222 and GSE126044 were used to explore the relationship between RUNX1 and treatment response [14–16]. According to the original response annotations, samples were mapped as responders (R) and nonresponders (NR). In each cohort, RUNX1 expression was compared between the R and NR groups, effect sizes and adjusted significance levels were calculated, and descriptive metrics including receiver operating characteristic (ROC) curves and response odds ratios were evaluated (Table S8). Progression‐free survival (PFS) information was available in GSE135222. Therefore, exploratory Kaplan–Meier and Cox analyses were performed. Because of the small sample sizes, limited number of events in the responder groups, and lack of key annotations such as driver mutations, TMB, PD‐L1, TIDE, and IPS, all ICI‐related analyses were restricted to exploratory purposes and were not used to validate the predictive value of RUNX1 for immunotherapy response.

### 2.7. Single‐Cell RNA‐Seq Projection Analysis

GSE207422 single‐cell data were used to evaluate the localization of RUNX1 signals and target projection across NSCLC cell compartments [17]. Cells were organized into major categories, including epithelial‐like cells, myeloid cells, CD4+ T cells, CD8+ T cells, other immune cells, and unassigned cells (Table S9). We calculated RUNX1 expression, the RUNX1‐positive fraction, RUNX1 target projection, and the expression context of candidate ligand/receptor genes. Candidate ligand‐receptor axes included IL6‐IL6R/IL6ST, TGFB1‐TGFBR1/2, CSF1‐CSF1R, CXCL8‐CXCR1/2, CCL2‐CCR2, and CD274‐PDCD1. This analysis was used only to provide expression‐based support for candidate TME/ligand–receptor contexts. It does not constitute CellChat/LIANA permutation‐based evidence and does not support conclusions regarding physical contact, functional communication or signal transmission, or causal enrichment.

### 2.8. Spatial Transcriptomic Corroboration Analysis

E‐MTAB‐13530 Visium spatial transcriptomic data were used to corroborate RUNX1 and selected TME‐supporting genes at the tissue level [18]. The present study reused genuine Visium inputs, spot‐level tables, section‐level summaries, quantitative summaries of all 40 sections, and quality‐control information from the legacy workspace. An all‐section gene summary, section‐level tumor‐versus‐adjacent contrasts, and patient‐paired deltas were generated in the current analysis workspace. The main figure presents a group‐level Visium expression summary across all groups, paired spatial deltas, and the scRNA‐seq/spatial claim boundary. The spatial analysis supports only section‐ and patient‐level transcript‐expression concordance and does not demonstrate cell type–specific coexpression or communication, physical contact, protein colocalization, or RUNX1‐mediated causal enrichment.

### 2.9. Integrated Model and Claim Ladder

**Figure 3:**
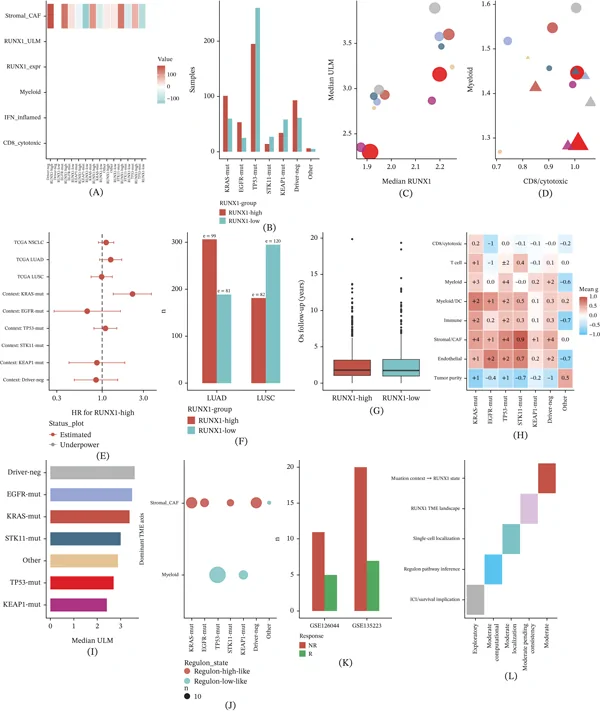
Mutation‐context‐aware integrative stratification framework for RUNX1–TME states. This figure integrates mutation context, RUNX1 expression/inferred target‐gene‐set state, TME effect summaries, exploratory survival/ICI evidence, and a claim ladder into a genotype‐context‐aware RUNX1–TME framework. (A) Integrated context matrix showing RUNX1 expression, RUNX1 ULM, CD8/cytotoxic, IFN/inflamed, myeloid, and stromal/CAF dimensions across driver context × RUNX1 group strata. (B) Joint group sample size showing sample numbers across driver contexts and RUNX1 groups. (C) Expression–target‐gene‐set state scatter plot comparing median RUNX1 expression and median RUNX1 ULM across contexts/groups. (D) TME phenotype space defined by CD8/cytotoxic and myeloid axes. (E) Subtype‐ and context‐specific OS Cox estimates comparing RUNX1 expression groups, including underpowered or model‐failed cases. (F) Survival data availability showing the sample numbers and event counts for RUNX1 groups in LUAD/LUSC. (G) OS follow‐up distribution stratified by RUNX1 group. (H) Figure 2 TME effect summary, providing a compressed display of the direction and support strength of multiple TME axes across different contexts. (I) Figure 6 target‐gene‐set activity ranking based on median RUNX1 ULM across driver contexts. (J) Hypothesis‐generating summary categorizing driver contexts by inferred target‐gene‐set state and dominant TME axis. (K) Exploratory ICI evidence summarizing the response sample composition in GSE135222/GSE126044. (L) Claim ladder summarizing the evidence levels for mutation context–associated RUNX1 state, RUNX1‐associated TME variation, single‐cell localization, computational target‐gene‐set/pathway inference, and exploratory ICI or survival implications. This figure proposes an integrated genotype‐context‐aware RUNX1–TME stratification framework. The survival and ICI results are hypothesis‐generating only and do not constitute clinical guidance, establish a stable ICI‐response predictor or an independent prognostic biomarker, or demonstrate causal TME remodeling or ligand–receptor communication. The complete framework has not been externally validated in an independent NSCLC cohort with fully matched molecular and clinical annotations.

Figure 3 summarizes mutation context, RUNX1 expression/target‐gene‐set state, and TME phenotype to construct a mutation context–aware RUNX1–TME stratification framework. The integrated matrix included dimensions such as RUNX1 expression, RUNX1 ULM, RUNX1 AUCell, CD8/cytotoxic, IFN/inflamed, antigen presentation, checkpoint, myeloid, and stromal/CAF axes. The hypothesis‐generating table summarized different driver contexts as combinations of RUNX1 state, target‐gene‐set state, and dominant TME axes. The claim ladder restricted the evidence throughout the manuscript to five levels: mutation context–associated RUNX1 state, RUNX1‐associated immune/stromal variation, single‐cell localization, computational target‐gene‐set inference, and hypothesis‐generating exploratory clinical association.

### 2.10. Statistical Analysis

Continuous variables were primarily compared using the Wilcoxon rank‐sum test. Hedges′s g was used as the effect‐size measure, Spearman correlation was used for correlation analysis, and the BH procedure was used for multiple‐testing correction. Survival analysis was performed using Kaplan–Meier curves and Cox proportional hazards models but only as an exploratory branch. Cohort‐specific sample sizes and event counts are summarized in Table S10; complete Cox model outputs are provided in Table S11; covariate availability and missingness are reported in Table S12; and the OS‐specific RUNX1 cutoff is defined in Table S13. Mutation context subgroups with small sample sizes or combined subgroups with *n* < 20 were labeled as underpowered and were not treated as strong statistical conclusions. All analyses were based on public datasets and traceable derived tables in the current workspace.

## 3. Results

### 3.1. Mutational Context Screening Nominates a RUNX1‐Associated Transcriptional State

We first avoided studying RUNX1 as a frequently mutated gene in NSCLC. Instead, we evaluated it within a framework designed to screen driver mutation context–associated transcriptional‐state candidates. Based on the TCGA‐LUAD/LUSC mutation–expression‐matched cohort, we defined prespecified driver mutation contexts, including EGFR, KRAS, TP53, STK11, KEAP1, and the prespecified driver‐context‐negative group, and assessed candidate transcriptional states within these genotype‐defined backgrounds.

Within the prespecified candidate set, RUNX1 ranked second according to the maximum absolute Hedges′s *g*, surpassed only by APOBEC3B. RUNX1 showed the strongest driver‐context effect in KEAP1‐mutant tumors, with a signed Hedges′s *g* of −0.422 and a BH‐adjusted *p* value of 3.52 × 10^−6^. APOBEC3B, as a mutation‐process proxy, showed the strongest overall context effect, whereas CYP1B1, as a smoking‐response proxy, showed a weaker effect. In an adjusted model incorporating driver context, tumor subtype, and TMB, STK11‐mutant and KEAP1‐mutant contexts retained coefficients associated with reduced RUNX1 expression (STK11‐mutant: coefficient = −0.132, BH‐adjusted *p* = 0.00144; KEAP1‐mutant: coefficient = −0.109, BH‐adjusted *p* = 7.16 × 10^−4^). The LUSC subtype was also associated with lower RUNX1 expression, whereas TMB did not independently explain the RUNX1 context effect in this model.

**Figure 4:**
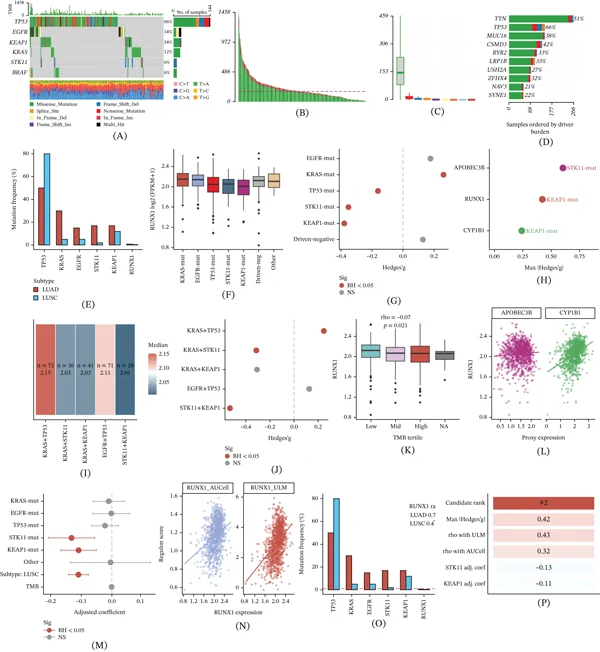
Mutation‐context screening nominates a RUNX1‐associated transcriptional state. This figure defines NSCLC driver mutation contexts using a mutation‐first strategy and performs a prespecified prioritization screen of candidate transcriptional states in the mutation–expression‐matched TCGA‐LUAD/LUSC cohort. (A) Oncoplot of selected NSCLC driver genes generated from GDC masked MAF files using maftools, showing mutation types and subtype annotations. (B) Sample‐level mutation‐burden waterfall showing mutation counts and nucleotide substitution classes. (C) Distribution of sample‐level mutation burden across driver‐burden/context categories. (D) Bar plot of the most frequently mutated genes ranked by sample count/frequency. (E) Mutation frequencies of selected driver genes and RUNX1 in LUAD and LUSC. (F) RUNX1 expression across driver mutation contexts. (G) Forest plot of Hedges′s *g* effect sizes for RUNX1 expression across single‐driver contexts. (H) Prioritization plot of prespecified candidate factors, including APOBEC3B, RUNX1, and CYP1B1, ranked by maximum absolute Hedges′s *g*. (I) Heatmap of median RUNX1 expression in selected comutation contexts, with sample sizes annotated. (J) Forest plot of Hedges′s *g* effect sizes for selected comutation contexts. (K) RUNX1 expression across TMB tertiles. (L) Scatter plots showing the relationships between RUNX1 expression and APOBEC3B or CYP1B1 proxy expression. (M) Candidate‐prioritization context‐model coefficients for RUNX1 expression after adjustment for cancer subtype and TMB. (N) Correlations between RUNX1 expression and the RUNX1 AUCell‐style or ULM target‐gene‐set readouts. (O) Subtype‐stratified mutation frequencies of selected driver genes and RUNX1. (P) Numerical summary of the RUNX1 candidate ranking and key supporting metrics. This figure supports the nomination of RUNX1 through prespecified mutation‐context candidate prioritization but does not support unbiased genome‐wide discovery, a RUNX1 mutation‐driver framing, or a causal conclusion that driver mutations directly activate RUNX1.

Furthermore, RUNX1 expression was significantly correlated with both inferred target‐gene‐set readouts. The Spearman rho between RUNX1 expression and the RUNX1 ULM score was 0.425 (BH‐adjusted *p* < 1 × 10^−16^), whereas the rho between RUNX1 expression and the AUCell‐style score was 0.319 (BH‐adjusted *p* < 1 × 10^−16^). These results support the inclusion of RUNX1 in subsequent analyses as a context‐associated transcriptional/target‐gene‐set state within the prespecified driver mutation‐context prioritization framework. Because RUNX1 itself is rarely mutated in NSCLC, all subsequent analyses treated RUNX1 as a downstream transcriptional interpreter associated with mutational background rather than as a mutation‐defined driver target (Figure 4).

### 3.2. Mutation‐Context Definition and Histology‐Aware Models Characterize RUNX1 Expression Associations

We next expanded the RUNX1 mutation‐context analysis across single‐driver contexts, comutation patterns, mutation burden, histologic strata, and covariate‐adjusted models. The six‐gene context denominator showed that the TP53‐mutant context was composed predominantly of LUSC samples, whereas histologic composition varied across the EGFR‐, KRAS‐, STK11‐, KEAP1‐, and the prespecified driver‐context‐negative group (Figure 1A). Accordingly, RUNX1 expression distributions were examined in the primary LUAD analysis, with LUSC presented separately as a descriptive sensitivity analysis (Figure 1B,C).

In the primary LUAD analysis, KRAS‐mutant tumors showed a trend toward higher RUNX1 expression, whereas STK11‐mutant and KEAP1‐mutant tumors showed lower RUNX1 expression distributions (Figure 1B). The KEAP1‐mutant context retained the clearest negative association in adjusted models. Context‐specific sample sizes were highly uneven in the descriptive LUSC analysis. The KRAS‐mutant, EGFR‐mutant, and STK11‐mutant groups contained few LUSC samples, whereas TP53‐mutant samples predominated. These sample‐size constraints limited the LUSC analysis to descriptive estimates of effect direction and uncertainty (Figure 1C).

The comutation layer further refined these associations. The six‐gene overlap matrix showed that driver labels were nonexclusive. TP53 mutations co‐occurred with EGFR, KRAS, KEAP1, and BRAF mutations in 56, 58, 88, and 39 samples, respectively, whereas KRAS mutations co‐occurred with STK11 and KEAP1 mutations in 36 and 34 samples, respectively (Figure 1D). The comutation burden panel also showed that a substantial proportion of samples carried mutations in more than one of the six context genes (Figure 1J). Consequently, comparisons between context‐positive samples and all remaining samples cannot be interpreted as strict mutant‐versus‐wild‐type contrasts. These comparisons were interpreted as context‐associated patterns without attributing independent causal effects to individual driver labels.

Adjusted context models further quantified these associations (Figure 1E). In the all‐NSCLC model, the STK11‐mutant context was associated with lower RUNX1 expression (*β* = −0.093, 95% CI = −0.160 to −0.026, BH FDR = 0.038). The KEAP1‐mutant context had a negative coefficient but did not meet the BH‐adjusted significance threshold (*β* = −0.060, 95% CI = −0.106 to −0.014, BH FDR = 0.051). The KRAS‐mutant context had a positive coefficient and likewise did not meet this threshold (*β* = 0.047, 95% CI = 0.007 to 0.087, BH FDR = 0.063). In the primary LUAD model, the KEAP1‐mutant context retained a clear negative association (*β* = −0.152, 95% CI = −0.212 to −0.092, BH FDR = 1.69 × 10^−5^), whereas the KRAS‐mutant context showed a positive association (*β* = 0.057, 95% CI = 0.016 to 0.099, BH FDR = 0.038). The STK11‐mutant coefficient was negative but did not meet the BH‐adjusted significance threshold (BH FDR = 0.054). Confidence intervals were wide for most context estimates in the descriptive LUSC models, particularly for the small KRAS‐mutant, EGFR‐mutant, and STK11‐mutant groups. These results were therefore interpreted as descriptive sensitivity findings.

At the mutation‐burden level, the relationship between RUNX1 expression and TMB was weak (Figure 1F). Overall, TMB provided a weaker continuous mutation‐burden dimension, whereas the most interpretable differences were derived from the driver context and comutation layers. RUNX1‐high/low grouping used the median log2‐transformed RUNX1 expression in the analysis‐eligible TCGA cohort as the cutoff, which was approximately 2.085. This cutoff was used only for exploratory grouping and visualization and was not interpreted as a clinical threshold (Figure 1G,H).

The driver‐negative definition audit included 992 samples. The stored annotation classified 154 samples as driver negative, whereas 165 met the recomputed context‐negative definition, with 11 discordant classifications between the stored and recomputed results (Figure 1I). Subsequent analyses used the prespecified six‐gene driver‐context‐negative operational group as an auditable reference group without interpreting it as a biologically homogeneous subtype. The model sample‐size audit and context‐specific median RUNX1 panel also reported the context and reference sample sizes (n_context/n_reference) and median RUNX1 expression for each context in all NSCLC, the primary LUAD analysis, and the descriptive LUSC analysis (Figure 1K,L). These summaries documented the sample‐size constraints underlying each model estimate.

Collectively, RUNX1 expression showed histology‐aware, comutation‐aware, and covariate‐adjusted associations with mutation context. These results indicate that RUNX1 expression differs across genotype‐defined contexts in NSCLC, but they neither require nor support the conclusion that driver mutations directly activate or suppress RUNX1 transcription (Figure 1). These analyses also did not support interpreting the prespecified driver‐context‐negative group as a biologically homogeneous molecular subtype.

### 3.3. Public ICI Cohorts Support Only Restricted Exploratory Analysis

We further evaluated whether RUNX1 could be explored in public NSCLC ICI cohorts. Two available cohorts provided response information: GSE135222 (*n* = 27; 7 R and 20 NR) and GSE126044 (*n* = 16; 5 R and 11 NR). In GSE135222, R had lower median RUNX1 expression than NR (4.288 vs. 4.675), but the difference was not significant (Hedges^′^s *g* = −0.374, *p* = 0.370). In GSE126044, R had higher median RUNX1 expression than NR (12.178 vs. 11.706), but the difference likewise did not reach significance after correction (Hedges^′^s *g* = 0.990, *p* = 0.221; crosscohort BH‐adjusted *p* = 0.370).

**Figure 5:**
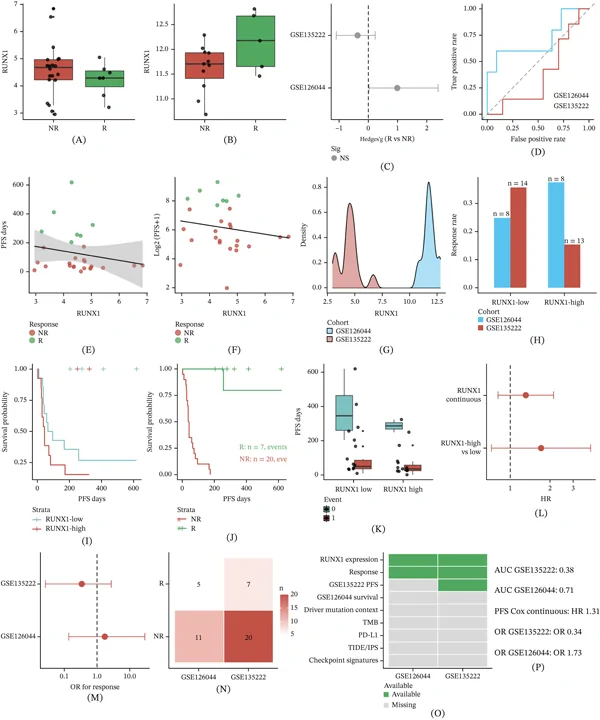
Restricted exploratory analysis of RUNX1‐response associations in public ICI cohorts. This figure uses GSE135222 and GSE126044 to evaluate the exploratory relationship between RUNX1 and NSCLC immune checkpoint inhibitor response while explicitly acknowledging limitations in sample size and data availability. (A) Comparison of RUNX1 expression between responders and nonresponders in GSE135222. (B) Comparison of RUNX1 expression between responders and nonresponders in GSE126044. (C) Summary of RUNX1 effect sizes for responders versus nonresponders across the two cohorts. (D) Cohort‐specific ROC curves and AUCs for RUNX1 in discriminating response status. (E) Scatter relationship between RUNX1 expression and PFS time in GSE135222, colored by response status. (F) Relationship between RUNX1 expression and log2(PFS + 1) in GSE135222, showing the joint distribution of PFS and response status. (G) Density distributions of RUNX1 expression across the two cohorts, indicating differences in expression range and sample composition between cohorts. (H) Response rate after stratification by RUNX1‐high/low groups, with sample sizes annotated for each group. (I) Kaplan–Meier curves for PFS stratified by RUNX1‐high/low groups in GSE135222. (J) Kaplan–Meier curves for PFS stratified by response status in GSE135222, with the numbers of responders/nonresponders and events annotated. (K) Distribution of PFS time and event status within RUNX1‐high/low groups. (L) Univariate PFS Cox regression results for RUNX1 modeled as both a continuous variable and a grouped variable in GSE135222. (M) Odds ratios for response associated with RUNX1‐high status across the two cohorts. (N) Sample‐size matrix for cohort × response status. (O) Data availability matrix for RUNX1 expression, response, cohort‐specific PFS/survival data, driver mutation context, TMB, PD‐L1, TIDE/IPS, and checkpoint signatures. (P) Exploratory numerical summary of AUC, PFS Cox regression, and response odds ratio results. The two cohorts showed directionally inconsistent RUNX1‐response associations and lacked key information on driver mutation context, TMB, PD‐L1, TIDE/IPS, and checkpoint signatures. This figure provides only a hypothesis‐generating presentation based on small public ICI cohorts and does not support RUNX1 as a stable or validated ICI biomarker, a clinical predictor of immunotherapy response, or validation of the mutation‐context‐aware RUNX1–TME framework in ICI‐treated cohorts.

The clinical and molecular annotations available in these public ICI cohorts limited the strength of the conclusions. Key information, including driver mutation labels, TMB, PD‐L1, TIDE, and IPS, was absent from the cohort files. Survival information supported an exploratory PFS branch only in GSE135222. Although the responder group in GSE135222 contained seven samples, only one PFS event was observed; therefore, the Kaplan–Meier curve could only be presented descriptively. ROC, odds ratio, and PFS analyses should all be interpreted as cohort‐specific exploratory summaries rather than validation of RUNX1 as an immunotherapy biomarker. The current data do not support the conclusion that RUNX1 predicts ICI response. Instead, they indicate that larger immunotherapy cohorts with driver mutation and treatment annotations are needed to further evaluate the mutation‐context RUNX1–TME framework (Figure 5).

### 3.4. Multialgorithm TME Scoring Reveals Mutation Context–Dependent RUNX1‐Immune/Stromal Associations

To assess whether RUNX1 state was associated with immune and stromal alterations, we reconstructed the TME landscape using four transcriptome‐based TME scoring approaches: MCP‐counter, xCell, ssGSEA immune signatures, and ESTIMATE. The merged analysis table contained 37,278 sample‐feature rows, covering four algorithm categories, 38 features, 15 TME axes, and six displayed mutation contexts. Among 228 context–feature comparisons between RUNX1‐high and RUNX1‐low groups, 57 met the BH‐supported and nonunderpowered criteria. Companion adjusted models using continuous RUNX1 were successfully estimated for all 228 feature–context combinations, with sample sizes ranging from 41 to 455. Of these, 67 RUNX1 coefficients met the BH FDR criterion. Figure 2A summarizes the high/low effects, whereas Figure 2B summarizes BH‐supported results from models adjusted, where applicable, for histology, TMB, estimated tumor purity, and comutation burden. Therefore, Figure 2 provides a multiaxis association map rather than a conclusion based on a single cell type or a single algorithm.

Within the high/low effect summaries, different driver contexts showed distinct RUNX1–TME patterns. In KRAS‐mutant tumors, the RUNX1‐high group showed higher MCP‐counter T‐cell signal (effect size = 0.481, BH‐adjusted *p* = 0.0252), higher myeloid/monocyte signal (effect size = 0.399, BH‐adjusted *p* = 0.0275), higher myeloid/DC signal (effect size = 0.734, BH‐adjusted *p* = 2.56 × 10^−4^), and higher CAF signal (effect size = 0.554, BH‐adjusted *p* = 0.0139). ESTIMATE immune and stromal scores were also higher (effect sizes = 0.528 and 0.843), whereas estimated tumor purity was lower (effect size = −0.736, BH‐adjusted *p* = 4.87 × 10^−4^). In TP53‐mutant tumors, RUNX1‐high samples showed higher scores across a broader range of immune/stromal axes, including T cell, B cell, myeloid, myeloid/DC, endothelial, and CAF‐related axes. EGFR‐mutant tumors exhibited a more mixed pattern, including lower MCP‐counter CD8/cytotoxic signal in the RUNX1‐high group (effect size = −0.855, BH‐adjusted *p* = 0.0243) and lower xCell CD4 memory T‐cell signal, together with higher endothelial and selected myeloid/DC axes. KEAP1‐mutant tumors showed higher scores for selected stromal/CAF‐related axes but no broad elevation across immune‐related scores.

These results support associations among mutation context, RUNX1 group, and multiaxis TME variation. The most consistently observed axes included stromal/CAF, myeloid/DC, endothelial, broad immune score, and tumor‐purity changes, whereas T‐cell/cytotoxic signals varied by context. Because outputs from CIBERSORT/CIBERSORTx, EPIC, quanTIseq, and TIMER were unavailable for the present analysis, these algorithms were not included in the evidence base. Because RUNX1 expression and all TME scores were derived from the same TCGA bulk RNA‐seq matrix, crossalgorithm concordance was interpreted as transcriptomic consistency and did not constitute independent immune validation. Figure 2 therefore supports RUNX1‐associated immune/stromal transcriptomic variation inferred from the available transcriptome‐based scoring methods, but it does not support causal RUNX1‐mediated immune remodeling (Figure 2).

### 3.5. Single‐Cell and Spatial Transcriptomics Localize RUNX1–TME Signals

**Figure 6:**
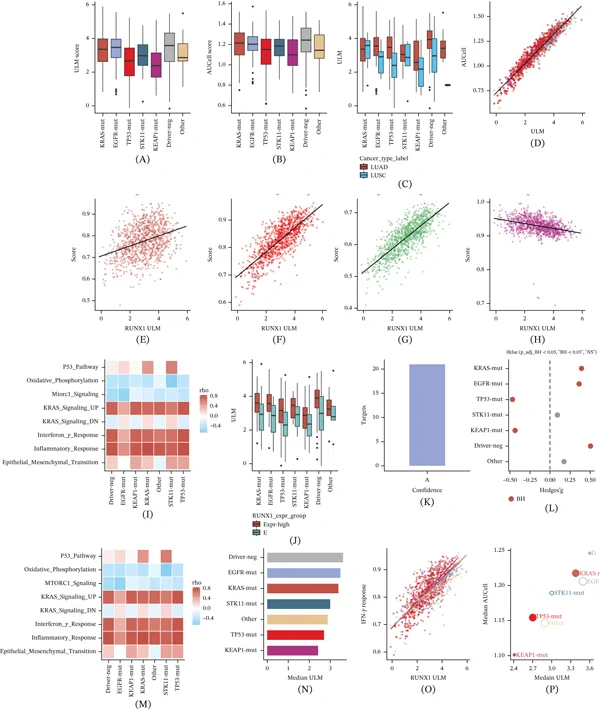
Inferred RUNX1 target‐gene‐set activity varies across driver mutation contexts. Based on DoRothEA/CollecTRI‐derived RUNX1 target information and ULM and AUCell‐style readouts, this figure evaluates inferred RUNX1 target‐gene‐set activity and its pathway associations. (A) Distribution of RUNX1 ULM target‐gene‐set scores across different driver contexts. (B) Distribution of RUNX1 AUCell‐style target‐gene‐set scores across different driver contexts. (C) Histology‐stratified comparison of RUNX1 ULM scores across contexts in LUAD and LUSC. (D) Sample‐level concordance between RUNX1 ULM and AUCell‐style readouts. (E) Association between RUNX1 ULM and the epithelial–mesenchymal transition score. (F) Association between RUNX1 ULM and the interferon‐gamma response score. (G) Association between RUNX1 ULM and the KRAS signaling up score. (H) Association between RUNX1 ULM and the oxidative phosphorylation score. (I) Context‐stratified heatmap of correlations between RUNX1 ULM and selected hallmark/pathway scores. (J) Distribution of RUNX1 ULM scores across RUNX1 expression groups within each driver context. (K) Number of confidence‐A RUNX1 targets included in the target set used for the bulk analysis. (L) Forest plot of Hedges′s *g* effect sizes for RUNX1 ULM across driver contexts, with BH‐supported associations indicated. (M) Target‐gene‐set readout × pathway × context heatmap showing the cross‐structure between inferred target‐gene‐set activity and immune, signaling, and metabolic pathways. (N) Driver contexts ranked by median RUNX1 ULM. (O) Relationship between RUNX1 ULM and the IFN‐*γ* response score. (P) Context‐level concordance between median RUNX1 ULM and median AUCell‐style readouts. This figure supports context‐dependent inferred RUNX1 target‐gene‐set activity and pathway associations. These expression‐based computational readouts do not establish direct regulation of RUNX1 by driver mutations, identify RUNX1 as a master regulator, or provide protein‐binding or perturbation‐based mechanistic evidence. Pathway scores were analyzed as precomputed values and were not recalculated after removing RUNX1 target genes. The pathway relationships are interpreted as correlations.

We projected RUNX1 expression and the RUNX1 target‐gene‐set information summarized in Figure 6 onto the GSE207422 NSCLC single‐cell dataset. This analysis included 92,330 cells, comprising 33,534 other immune cells, 16,317 myeloid cells, 11,546 CD4+ T cells, 11,430 CD8+ T cells, 9670 epithelial‐like cells, and 9833 unassigned cells. RUNX1 expression was detectable across multiple compartments, with the highest mean log2 expression observed in myeloid cells and epithelial‐like cells (myeloid mean = 0.630; epithelial − like mean = 0.561). The RUNX1‐positive fraction in epithelial‐like cells was 41.7%, yielding 4032 RUNX1‐high epithelial‐like cells for subsequent analysis of candidate ligand expression contexts.

The RUNX1 target projection used 18 detected confidence‐A signed RUNX1 targets from DoRothEA and was interpreted as an inferred target projection rather than evidence from a complete pySCENIC regulon analysis. This projection localized RUNX1‐associated target‐gene information to immune and epithelial‐like compartments, providing cellular‐level support for the bulk target‐gene‐set findings. Marker and target dot plots further showed that RUNX1 and selected target genes were not restricted to a single cell category, which is more consistent with a crosscompartment transcriptional‐state interpretation.

To explore potential epithelial‐to‐TME expression contexts, we compared ligand expression between RUNX1‐high and RUNX1‐low epithelial‐like cells and assessed receptor expression in annotated TME compartments. Multiple expression‐supported axes were retained, including IL6‐IL6R/IL6ST, TGFB1‐TGFBR1/2, CSF1‐CSF1R, CXCL8‐CXCR1/2, and CD274‐PDCD1. These results generated candidate ligand‐receptor expression contexts for RUNX1‐high epithelial‐like cells, but they do not constitute CellChat/LIANA‐based communication inference or evidence of physical contact, functional ligand‐receptor signaling, or altered immune‐cell abundance.

**Figure 7:**
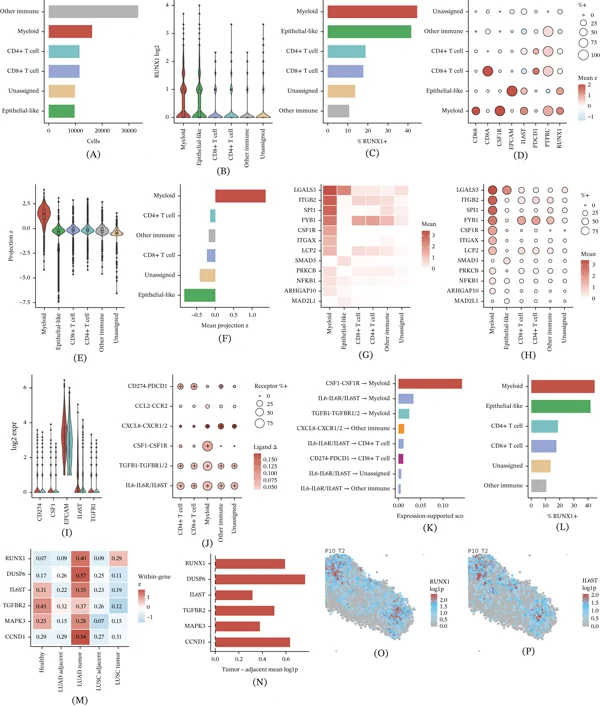
Single‐cell and spatial transcriptomics localize RUNX1‐associated candidate TME expression contexts. This figure integrates GSE207422 scRNA‐seq data and E‐MTAB‐13530 Visium data to localize RUNX1 expression, an inferred DoRothEA‐derived RUNX1 target‐gene‐set projection, and candidate epithelial‐like‐to‐TME ligand–receptor expression contexts. (A) Cellular composition of major cell‐type compartments in GSE207422. (B) Distribution of RUNX1 expression across different cell types. (C) RUNX1‐positive fraction across different cell types. (D) Marker and candidate‐gene dot plot showing CD68, CD8A, CSF1R, EPCAM, IL6ST, PDCD1, PTPRC, and RUNX1. (E) Distribution of the inferred RUNX1 target‐gene‐set projection score across different cell types. (F) Mean RUNX1 target‐gene‐set projection score for each cell type. (G) Heatmap of selected RUNX1 target‐set genes across major cell types. (H) Detection dot plot of the selected RUNX1 target‐set genes. (I) Expression distributions of EPCAM and selected candidate genes in RUNX1‐high epithelial‐like cells. (J) Candidate ligand–receptor expression‐support bubble plot for prespecified axes, including IL6–IL6R/IL6ST, TGFB1–TGFBR1/2, CSF1–CSF1R, CXCL8–CXCR1/2, CCL2–CCR2, and CD274–PDCD1. (K) Expression‐support scores for the top ligand–receptor candidates. (L) RUNX1‐positive fraction across different cell‐state compartments. (M) Section/group‐level spatial expression summary of RUNX1 and selected TME‐supporting genes in E‐MTAB‐13530 Visium data. (N) Tumor‐minus‐adjacent differences in the mean log1*p* expression of selected genes in matched LUAD tumor and adjacent sections. (O) Visium spot map of RUNX1 expression in a selected LUAD tumor section. (P) Visium spot map of IL6ST expression in the same section. This figure supports the localization of RUNX1 expression and the inferred target‐gene‐set projection across scRNA‐seq compartments, together with tissue‐level transcript‐expression patterns of RUNX1 and selected TME‐supporting genes in Visium sections. The epithelial‐like annotation does not establish malignant identity. The target‐gene‐set projection does not constitute a de novo or complete pySCENIC regulon reconstruction, and the ligand–receptor panels do not provide CellChat/LIANA permutation‐based evidence. The spatial results do not demonstrate cell type–specific coexpression, functional communication, physical cell–cell contact, protein colocalization, or direct immune‐cell recruitment by RUNX1.

We then incorporated a spatial transcriptomic layer using E‐MTAB‐13530 NSCLC Visium sections to provide tissue‐level context. Group‐level spatial summaries and matched LUAD tumor‐adjacent comparisons showed differences between tumor and adjacent sections in RUNX1 and selected TME‐supporting genes, including IL6ST and DUSP6. Spot‐level maps from a selected pair were presented only as visual examples of the distributions of RUNX1 and candidate TME‐supporting genes within the tissue sections. These spatial analyses provided tissue‐level transcript‐expression concordance for RUNX1 and selected TME‐supporting genes but did not establish cell type–specific coexpression, physical cell–cell contact, protein colocalization, or functional ligand‐receptor signaling (Figure 7).

### 3.6. Inferred RUNX1 Target‐Gene‐Set Activity Varies Across Driver Mutation Contexts

We further examined whether inferred RUNX1 target‐gene‐set activity varied by mutation context. The ULM‐based RUNX1 target‐gene‐set score was highest in the prespecified driver‐context‐negative group, followed by EGFR‐mutant, KRAS‐mutant, STK11‐mutant, other, TP53‐mutant, and KEAP1‐mutant tumors. The prespecified driver‐context‐negative group showed the strongest positive ULM shift (median = 3.603; delta = 0.672; Hedges^′^s *g* = 0.505; BH − adjusted *p* = 4.57 × 10^−8^). EGFR‐mutant and KRAS‐mutant tumors also showed higher ULM activity than their respective reference samples (EGFR‐mutant: median = 3.494; delta = 0.525; BH − adjusted *p* = 0.00198; KRAS‐mutant: median = 3.382; delta = 0.452; BH − adjusted *p* = 1.89 × 10^−5^). In contrast, TP53‐mutant and KEAP1‐mutant tumors showed lower ULM activity (TP53‐mutant: median = 2.702; delta = −0.577; BH − adjusted *p* = 6.93 × 10^−12^; KEAP1‐mutant: median = 2.410; delta = −0.651; BH − adjusted *p* = 1.18 × 10^−4^).

The AUCell‐style score showed a partially concordant pattern. The prespecified driver‐context‐negative group and KRAS‐mutant tumors ranked higher, whereas TP53‐mutant and KEAP1‐mutant tumors ranked lower and remained significant after correction. EGFR‐mutant tumors showed higher ULM scores, but the AUCell‐style score did not reach significance, indicating that concordance across different inference readouts was more stable for the prespecified driver‐context‐negative group and the KRAS‐mutant, TP53‐mutant, and KEAP1‐mutant contexts. This discrepancy helps avoid overinterpreting a single scoring method as a definitive regulatory readout.

Pathway correlation analysis showed that RUNX1 ULM was positively correlated with inflammatory response, interferon‐gamma response, and KRAS signaling up in multiple contexts, including KRAS‐mutant, TP53‐mutant, STK11‐mutant, KEAP1‐mutant, and the prespecified driver‐context‐negative group. RUNX1 ULM was also negatively correlated with oxidative phosphorylation in several contexts and was associated with mTORC1 signaling in selected contexts. Overall, Figure 6 supports context‐dependent inferred RUNX1 target‐gene‐set activity and its pathway associations, but these findings remain computational target‐gene‐set and pathway associations rather than protein‐level or perturbation‐based experimental evidence (Figure 6).

### 3.7. Integrated Model Summarizes a Mutation‐Context‐Aware RUNX1–TME Framework

Finally, we integrated mutation context, RUNX1 expression/target‐gene‐set state, and TME phenotype into a genotype‐context‐aware RUNX1–TME framework. The integrated matrix summarized differences between RUNX1‐high and RUNX1‐low groups across different driver contexts along axes including RUNX1 expression, RUNX1 ULM, RUNX1 AUCell, CD8/cytotoxic, IFN/inflamed, antigen presentation, checkpoint, myeloid, and stromal/CAF. This matrix showed that RUNX1‐high groups are not equivalent across different driver backgrounds. KRAS‐mutant and TP53‐mutant RUNX1‐high tumors exhibited stronger immune/stromal shifts, whereas EGFR‐mutant RUNX1‐high tumors showed a more mixed T‐cell/cytotoxic profile. KEAP1‐mutant tumors had overall lower RUNX1 expression and inferred target‐gene‐set activity, primarily retaining selected myeloid and stromal features without broad elevation across immune‐related transcriptomic scores.

The survival branch was interpreted conservatively. In the overall TCGA NSCLC cohort, RUNX1‐low status was not significantly associated with overall survival in the exploratory analysis (OS; HR = 1.11, 95% CI 0.91–1.37, *p* = 0.305). Analyses stratified by LUAD and LUSC subtypes likewise did not reach significance. A context‐specific signal was observed in KRAS‐mutant tumors, in which RUNX1‐low status was associated with worse OS (HR = 2.25, 95% CI 1.34–3.76, *p* = 0.00208; BH − adjusted *p* = 0.0167). Other contexts did not show survival support of comparable strength, and the survival grouping for STK11‐mutant tumors was underpowered. Therefore, the survival results should be regarded as a context‐specific exploratory signal rather than evidence that RUNX1 is a general prognostic biomarker in NSCLC.

The hypothesis‐generating table further summarized different driver contexts into hypothesis‐generating genotype‐context groups. KRAS‐mutant and EGFR‐mutant contexts were summarized as RUNX1‐high‐like/target‐gene‐set‐high‐like states accompanied by stromal/CAF‐associated TME features. TP53‐mutant and KEAP1‐mutant contexts were summarized as RUNX1‐low‐like/target‐gene‐set‐low‐like states accompanied by myeloid‐associated features. The prespecified driver‐context‐negative group showed a RUNX1‐low‐like but target‐gene‐set‐high‐like state. Because the driver‐context labels were nonexclusive, these summaries describe overlapping context‐associated patterns and do not define mutually exclusive molecular subtypes. The claim ladder restricted the final interpretation to five levels of evidence: mutation context–associated RUNX1 state, RUNX1‐associated immune/stromal variation, cellular localization of RUNX1 signal, inferred context‐dependent target‐gene‐set activity, and hypothesis‐generating exploratory clinical association. This integrated model supports a mutation‐context‐aware RUNX1–TME stratification framework, rather than clinical guidelines, a stable predictor of ICI response, or a proven causal remodeling mechanism (Figure 3).

## 4. Discussion

This study does not attempt to redefine RUNX1 as a frequently mutated driver gene in NSCLC. Instead, it places RUNX1 in a more complex and often overlooked position: a transcriptional/target‐gene‐set state interpreter operating downstream of driver mutation context. Previous TCGA and cBioPortal studies have demonstrated the substantial genomic heterogeneity of LUAD and LUSC while also showing that RUNX1 is not a frequent mutational event in NSCLC [1, 2, 10, 11]. This distinction is central to the logic of the present study. Because the mutation frequency of RUNX1 itself is low, the rationale for this study would be difficult to establish within a “mutation driver” framework. However, when the question is reframed as whether different mutational backgrounds shape distinct downstream transcriptional states, RUNX1 becomes an interpretable node. In other words, the central issue is not whether RUNX1 initiates NSCLC, but whether a RUNX1‐associated candidate transcriptional state can help organize and explain TME differences across distinct genotype contexts. This positioning lowers the strength of mechanistic claims but makes the research question more consistent with the real heterogeneity of NSCLC.

An important alternative explanation must be considered: The association between RUNX1 and driver context may not arise from specific regulation, but instead from the combined effects of LUAD/LUSC composition, tumor purity, smoking‐related expression [29], TMB, or differences in cellular composition. To address several of these alternative explanations, the study incorporated histology‐aware comparisons, mutation‐burden analyses, and context models adjusted for histology where applicable, TMB, estimated tumor purity, and six‐gene comutation burden. However, these adjustments cannot fully exclude residual confounding. In particular, within nonmutually exclusive driver labels and comutation groups, the same sample may contribute to multiple contexts, making effect sizes more appropriately interpreted as context‐associated shifts rather than independent mutational effects. Therefore, the most cautious interpretation of Figures 4 and 1 is that RUNX1 state undergoes detectable shifts across genotype‐defined contexts, rather than that a specific driver mutation directly upregulates or downregulates RUNX1.

The value of the expanded mutation‐context analysis lies in the fact that it does not reduce all NSCLC cases to a single direction of effect. In the primary LUAD model, the KRAS‐mutant context was associated with higher RUNX1 expression, whereas the KEAP1‐mutant context showed a clear negative association and the STK11‐mutant context showed a negative trend. Estimates in the descriptive LUSC analysis were less precise because context‐specific sample sizes were small and uneven. The six‐gene overlap matrix and comutation burden analysis further showed that driver labels were nonexclusive, precluding clean attribution of these patterns to individual mutations. This heterogeneity may appear to weaken the narrative of a single RUNX1 mechanism, but it is biologically more plausible. Previous studies of KRAS, STK11, KEAP1, and TP53 have shown that these mutational backgrounds correspond to distinct immune profiles and therapeutic vulnerabilities [3–7]. RUNX1 may not represent a universal oncogenic axis acting in the same direction across all NSCLC backgrounds. Rather, it may reflect a context‐dependent transcriptional state shaped jointly by mutational ecology, histologic subtype, metabolic status, and immune pressure. For this reason, the present study should not seek an overly simplified linear model but should instead preserve the biological tension among different driver contexts.

The TME results also require a clear distinction between the presence of a signal and the establishment of a mechanism. MCP‐counter, xCell, ssGSEA, and ESTIMATE collectively suggested context‐dependent variation between RUNX1‐high and RUNX1‐low tumors across stromal/CAF, myeloid/DC, endothelial, immune score, tumor purity, and selected T‐cell/cytotoxic axes [24–26, 28]. However, bulk deconvolution and signature scoring are essentially expression‐pattern decomposition methods. They cannot automatically distinguish true changes in cell proportions, changes in cell states, tumor purity variation, or stromal admixture. In particular, CAF, endothelial, and tumor purity axes may be interdependent, whereas myeloid/DC axes may also be influenced by tissue sampling and inflammatory background. Therefore, the significance of Figure 2 is not that it proves RUNX1‐mediated immune–stromal remodeling, but that it provides a TME phenotype map showing concordant associations across the available scoring methods applied to the same bulk RNA‐seq matrix, which can help guide subsequent single‐cell, spatial, and experimental validation.

This also explains why the present study does not present any single immune cell type as the core conclusion. Selecting only the most significant individual feature could easily produce an oversimplified narrative in which RUNX1‐high status corresponds to an increase in a particular cell type. However, the current data more strongly support multiaxis variation in transcriptome‐derived TME scores, and the directions of T‐cell/cytotoxic, myeloid, CAF, and purity‐related score differences are not fully consistent across different mutation contexts. Preserving this multiaxis structure is more faithful to the data than forcing the findings into a single immune axis. Accordingly, the conclusion of Figure 2 should be interpreted as RUNX1‐associated immune/stromal transcriptomic variation rather than RUNX1‐driven immune remodeling.

The single‐cell and spatial layers improve the tissue‐level interpretability of the framework, but they do not eliminate causal uncertainty. GSE207422 showed that RUNX1 signal and RUNX1 target projection could be localized to epithelial‐like, myeloid, and other immune compartments [17], and RUNX1‐high epithelial‐like cells also exhibited several expression‐supported candidate ligand‐receptor expression axes. These findings suggest that the RUNX1‐associated state may reside at an expression interface between tumor epithelium and the TME. However, the current analysis did not include cell type–resolved perturbation, CellChat/LIANA‐based communication inference, or receptor‐ligand colocalization at the spatial protein level. Therefore, axes such as IL6/IL6R/IL6ST, TGFB1/TGFBR1/2, CSF1/CSF1R, CXCL8/CXCR1/2, CCL2/CCR2, and CD274/PDCD1 should be regarded as candidate ligand‐receptor expression contexts rather than established communication mechanisms.

The E‐MTAB‐13530 Visium data provided additional tissue‐level context while also introducing another set of limitations [18]. Section‐level summaries and patient‐paired tumor‐adjacent comparisons showed differences in RUNX1 and selected TME‐supporting gene expression, whereas spot‐level maps were presented only as visual examples of their transcript distributions within tissue sections. However, each Visium spot contains a mixture of multiple cells, and these analyses cannot resolve specific cellular contact, cell type–specific coexpression, secretion directionality, protein colocalization, or causal chains. The spatial results therefore place bulk‐ and scRNA‐seq‐derived inferences back into tissue architecture as a biological plausibility assessment, but they do not replace spatial proteomics, multiplex immunofluorescence, or in situ functional validation.

The RUNX1 target‐gene‐set analysis further strengthens the concept of a context‐dependent state, but its mechanistic interpretation must also remain cautious. ULM and AUCell‐style readouts supported changes in inferred RUNX1 target‐gene‐set activity across driver contexts and showed associations with programs such as inflammatory response, IFN‐*γ* response, KRAS signaling up, and oxidative phosphorylation. These readouts depend on prior target‐gene‐set information and expression matrix‐based inference frameworks [20–23]. However, they are expression‐based inferences derived from predefined target sets and are not equivalent to evidence that RUNX1 binds the promoters or enhancers of these genes in the tumor samples analyzed here. ULM and AUCell were concordant in selected contexts, but they did not completely overlap across all contexts, indicating that target‐gene‐set inference itself is influenced by target‐set definition, expression dynamic range, and sample composition. Therefore, Figure 6 supports computational target‐gene‐set and pathway associations, but it does not establish RUNX1 as a master regulator or demonstrate a protein‐level mechanism.

From a clinical translation perspective, Figures 5 and 3 are the most prone to misinterpretation. The public ICI cohorts had small sample sizes, lacked key annotations such as driver mutation labels, TMB, PD‐L1, and TIDE/IPS, and contained very few PFS events in the responder group of GSE135222 [14–16]. Therefore, even if certain panels show directional differences, they cannot be interpreted as evidence that RUNX1 predicts ICI response. Previous studies have already shown that STK11, KEAP1, KRAS, and TP53 backgrounds can influence the immune ecology and clinical outcomes associated with PD‐1/PD‐L1 therapy [6, 7]. Similarly, the TCGA survival branch did not support RUNX1 as a general prognostic factor. Instead, it suggests that the clinical significance of RUNX1 may depend on specific mutation contexts and currently remains hypothesis‐generating. This negative finding defines a clear boundary for future work: Evaluation requires immunotherapy cohorts annotated for mutation status, treatment, and TME features rather than continued testing of a single‐gene biomarker in small, sparsely annotated ICI datasets.

External matched validation remains a major limitation of the current framework. No independent non‐TCGA NSCLC cohort with matched mutation, expression, histology, TMB, estimated tumor purity, treatment, and TME annotations was available to evaluate the complete mutation‐context RUNX1–TME framework. Some public LUAD expression cohorts may provide limited directional context, but they cannot be generalized to LUSC or pan‐NSCLC and cannot validate the complete six‐gene context structure and its prespecified driver‐context‐negative group, TMB/purity‐adjusted analyses, or ICI response. Therefore, the TCGA‐derived framework remains discovery‐level and hypothesis‐generating. Full external validation will require independent, comprehensively annotated NSCLC cohorts.

Thus, the main contribution of this study is not the presentation of a closed mechanistic chain, but the proposal of a falsifiable stratification hypothesis: NSCLC driver mutation contexts may correspond to distinct TME phenotypes through different RUNX1 expression/target‐gene‐set states. This correspondence is supported at multiple, but noncausal, levels, including bulk scoring, single‐cell localization, spatial concordance, and computational target‐gene‐set inference. This hypothesis generates several predictions that can be tested in future experiments. First, the effects of RUNX1 perturbation on immune/stromal gene programs should differ among KRAS‐mutant, STK11/KEAP1‐mutant, and TP53‐mutant models. Second, the IL6ST/TGFB/CSF1/CXCL8/CCL2/CD274‐related expression axes in RUNX1‐high epithelial‐like cells should show corresponding tissue‐neighborhood relationships in spatial proteomics or multiplex immunofluorescence. Third, clinically meaningful stratification should not rely on RUNX1 expression alone but should jointly incorporate driver context, RUNX1 target‐gene‐set readouts, and TME phenotype.

This study has several clear limitations. First, the main evidence was derived from public bulk transcriptomic data and computational inference and lacks orthogonal wet‐lab validation. Second, sample sizes were imbalanced across mutation context subgroups, and selected STK11, KEAP1, or comutation subgroups had limited statistical power. Third, nonmutually exclusive driver labels made it difficult to fully decompose context effects into single‐mutation effects. Fourth, the single‐cell and spatial analyses provided localization and tissue‐level context but did not include cell type–specific communication inference, spatial‐neighborhood analysis, or functional perturbation validation. Fifth, the ICI cohorts had small sample sizes and insufficient annotations and could only be used for restricted exploratory analysis. Sixth, no independent NSCLC cohort with matched mutation, expression, histology, TMB, estimated tumor purity, treatment, and TME annotations was available to validate the complete framework. Seventh, the primary TME analysis was restricted to the available MCP‐counter, xCell, ssGSEA, and ESTIMATE outputs, and its findings cannot be extrapolated to other algorithms not included in the analysis.

In summary, this study proposes a mutation‐context‐aware RUNX1–TME stratification framework that positions RUNX1 as a transcriptional/target‐gene‐set state interpreter downstream of NSCLC driver mutation context, rather than as an independent mutation driver, a universal prognostic factor, or a validated predictor of immunotherapy response. The value of this framework lies in organizing inconsistent mutation contexts, RUNX1 states, and multiaxis TME variation into a testable model. Its limitations are also clear: The current evidence mainly supports association, localization, and computational inference. Future work should use mutation‐stratified perturbation, spatial protein validation, functional coculture assays, and more comprehensively annotated ICI cohorts to test whether RUNX1 truly participates in linking genotype context with TME niche heterogeneity.

## Author Contributions

J.W. conceived the study, performed the main analyses, interpreted the data, and drafted the manuscript. S.L. contributed to data collection, literature review, and data interpretation. J.Z. assisted with methodology, validation, and manuscript revision. J.Y. contributed to project coordination, critical discussion, and manuscript revision. H.M. supervised the study, provided overall guidance, critically revised the manuscript, and approved the final version.

## Funding

This study was funded by the Liaoning Provincial Science and Technology Plan Joint Program (Natural Science Foundation–Doctoral Research Start‐up Project), Grant No. 2024‐BSLH‐056.

## Disclosure

All authors read and approved the final manuscript.

## Ethics Statement

This study used only publicly available, deidentified datasets. No new human samples, animal experiments, or clinical interventions were involved. Therefore, additional ethical approval and informed consent were not required.

## Conflicts of Interest

The authors declare no conflicts of interest.

## Supporting Information

Additional supporting information can be found online in the Supporting Information section.

## Supporting information


**Supporting Information 1** Table S1: Molecular, mutation context, and microenvironmental variables at the sample level in the TCGA NSCLC cohort. The table lists TCGA sample identifiers, histologic subtype, stored and recomputed six‐gene driver‐context‐negative status, the number of mutated prespecified context genes, binary mutation indicators for EGFR, KRAS, TP53, STK11, KEAP1, BRAF, and RUNX1, RUNX1 expression as FPKM and log2(FPKM +1), ESTIMATE‐derived tumor purity and immune, stromal, and ESTIMATE scores, and inferred RUNX1 ULM and AUCell‐style target‐gene‐set scores. Binary indicators are coded as 1 for present and 0 for absent. RUNX1 mutation status was not used to define the driver‐context‐negative group.

**Supporting Information 2** Table S2: Quality‐control audit of the driver‐context‐negative definition in the TCGA NSCLC cohort. The stored driver‐context‐negative annotation was compared with a classification recomputed from the absence of detected mutations in EGFR, KRAS, TP53, STK11, KEAP1, and BRAF across 992 samples. The table reports the total cohort size, the number classified as driver‐context negative using each approach, and the number of discordant assignments.

**Supporting Information 3** Table S3: Descriptive molecular profile of the stored driver‐context‐negative operational group in TCGA NSCLC. The table summarizes sample counts overall and by histology, the cohort‐wide RUNX1 expression cutoff, RUNX1‐high and RUNX1‐low counts, ESTIMATE‐derived tumor purity and immune, stromal, and composite scores, inferred RUNX1 ULM and AUCell‐style target‐gene‐set scores, and mutation‐positive sample counts in the stored driver‐context‐negative and nondriver‐context‐negative groups. Values are medians or sample counts as indicated. RUNX1 mutation status was reported separately and was not used to define the group.

**Supporting Information 4** Table S4: Distribution of RUNX1‐high and RUNX1‐low samples by histology and stored driver‐context category in TCGA NSCLC. Sample counts are reported separately for LUAD and LUSC across the KRAS‐mutant, EGFR‐mutant, TP53‐mutant, STK11‐mutant, KEAP1‐mutant, driver‐context‐negative, and other categories. RUNX1‐high and RUNX1‐low groups were defined using the median log2‐transformed RUNX1 expression of approximately 2.085 in the analysis‐eligible TCGA cohort. The stored driver‐context categories represent the mutually exclusive display assignments used in this analysis. The assignment hierarchy for comutated tumors and the definition of the other category are described in Section 2.2.

**Supporting Information 5** Table S5: Associations between stored driver‐context categories and RUNX1 expression after covariate adjustment in TCGA NSCLC. Linear models were fitted in the complete NSCLC cohort and separately in LUAD and LUSC. The pooled NSCLC models included histology, continuous tumor mutational burden, estimated tumor purity, and six‐gene comutation burden as covariates. Histology was omitted from the LUAD‐ and LUSC‐specific models. The *β* coefficient and its 95% confidence interval represent the context coefficient relative to the remaining samples. The variables n_context and n_reference report context and reference counts before complete‐case filtering, whereas *n* reports the number included in the fitted model. LUSC‐specific estimates were treated as descriptive sensitivity results.

**Supporting Information 6** Table S6: Associations between continuous RUNX1 expression and transcriptome‐derived immune and stromal features across driver‐context groups. Feature‐level linear models were fitted for 38 features derived from MCP‐counter, xCell, single‐sample gene set enrichment analysis, and ESTIMATE across the displayed driver‐context groups. The model was specified as score ~ RUNX1_log2 + cancer_type_label + TMB + Estimated_purity_cov + co_mutation_count. The table reports the RUNX1 regression coefficient, 95% confidence interval, nominal *p* value, Benjamini–Hochberg‐adjusted *p* value, estimation status, and complete‐case sample size for each context–feature combination. Positive and negative coefficients indicate higher and lower feature scores with increasing log2‐transformed RUNX1 expression, respectively.

**Supporting Information 7** Table S7: Axis‐level summary of continuous RUNX1 and tumor microenvironment association models across driver‐context groups. For each driver‐context group and tumor microenvironment axis, *β* represents the arithmetic mean of the feature‐level coefficients reported in Table S6. The variable n_estimated indicates the number of successfully fitted feature‐level models, and n_BH indicates the number of features with a Benjamini–Hochberg‐adjusted *p* value below 0.05. Because the feature scores were generated by different algorithms and are expressed on algorithm‐specific scales, the mean coefficients provide directional summaries and are not directly comparable in magnitude across axes.

**Supporting Information 8** Table S8: Exploratory associations between RUNX1 expression and immune checkpoint inhibitor response. For GSE126044 and GSE135222, the table reports the total numbers of responders and nonresponders, median RUNX1 expression by response status, Hedges′s *g* for responders versus nonresponders, Wilcoxon rank‐sum *p* values, Benjamini–Hochberg‐adjusted *p* values, and Fisher exact odds ratios with 95% confidence intervals for response in the RUNX1‐high versus RUNX1‐low groups. Positive Hedges′s *g* values indicate higher RUNX1 expression among responders. RUNX1 expression values are presented on cohort‐specific normalized scales and should not be compared directly across cohorts. The RUNX1‐high and RUNX1‐low groups were defined using the cutoff rule specified in Section 2.6. All analyses were exploratory.

**Supporting Information 9** Table S9: Single‐cell compartment summary of RUNX1 expression and epithelial and immune marker context in GSE207422. The table reports the number of cells, mean log2‐transformed RUNX1 expression, percentage of RUNX1‐positive cells, and mean log2‐transformed EPCAM and PTPRC expression across six major cell compartments. The epithelial‐like designation represents a major‐compartment annotation based on marker‐expression context; definitive malignant‐cell identification would require copy‐number variation inference or original tumor‐cell labels. The evidence fields summarize the available annotation evidence, remaining evidence gap, and permitted interpretation.

**Supporting Information 10** Table S10: Sample size and event audit for exploratory overall survival analyses. The table reports the numbers of cases with available overall survival data, overall survival events, cases and events in the global median‐defined RUNX1‐high and RUNX1‐low groups, events per variable for three‐ and five‐covariate specifications, and the underpowered flag for the overall TCGA NSCLC cohort, LUAD and LUSC, and the listed mutation‐context subsets. Full adjustment was classified as underpowered when the five‐covariate events‐per‐variable value was below 10.

**Supporting Information 11** Table S11: Cox proportional hazards model outputs for exploratory overall survival analyses. For TCGA NSCLC, LUAD, LUSC, and the listed mutation‐context subsets, the table reports univariable models using grouped or continuous RUNX1 and adjusted models containing the indicated clinical, tumor mutational burden, and estimated tumor purity covariates. The minimally adjusted clinical models included age, sex, and stage, with histology additionally included in the pooled NSCLC analysis. Context‐specific minimal models included age and sex. Expanded models additionally included tumor mutational burden and estimated tumor purity. Each row reports the model term, sample size, event count, hazard ratio, 95% confidence interval, nominal *p* value, applicable Benjamini–Hochberg‐adjusted *p* value, events‐per‐variable audit, estimation status, and interpretation category. In grouped models, RUNX1‐high was the reference category. In continuous models, the hazard ratio represents a one‐unit increase in RUNX1 log2(FPKM + 1). All survival analyses were exploratory.

**Supporting Information 12** Table S12: Covariate availability and missingness audit for exploratory survival analyses. For the overall TCGA NSCLC cohort, LUAD and LUSC, and the listed mutation‐context subsets, the table reports the total, available, and missing sample counts for overall survival status and follow‐up time, RUNX1 expression and expression group, age, sex, stage, histology, tumor mutational burden, estimated tumor purity, mutation indicators, and pack‐years smoked.

**Supporting Information 13** Table S13: Global median RUNX1 cutoff used in exploratory overall survival analyses. The global median RUNX1_log2 value was calculated among 971 TCGA NSCLC cases with available overall survival data and 382 observed events. RUNX1‐high was defined as RUNX1_log2 greater than or equal to 2.084988, and RUNX1‐low was defined as RUNX1_log2 below 2.084988. Continuous RUNX1 models were also evaluated.

## Data Availability

All datasets analyzed in this study are publicly available from TCGA/GDC, GEO, and ArrayExpress, including TCGA‐LUAD/LUSC, GSE135222, GSE126044, GSE207422, and E‐MTAB‐13530. Derived data are available from the corresponding author upon reasonable request.
